# Codon-optimization in gene therapy: promises, prospects and challenges

**DOI:** 10.3389/fbioe.2024.1371596

**Published:** 2024-03-28

**Authors:** Anastasiia Iu Paremskaia, Anna A. Kogan, Anastasiia Murashkina, Daria A. Naumova, Anakha Satish, Ivan S. Abramov, Sofya G. Feoktistova, Olga N. Mityaeva, Andrei A. Deviatkin, Pavel Yu Volchkov

**Affiliations:** ^1^ Federal Research Center for Innovator and Emerging Biomedical and Pharmaceutical Technologies, Moscow, Russia; ^2^ The MCSC named after A. S. Loginov, Moscow, Russia

**Keywords:** gene therapy, codon-optimization metrics, mRNA, immunogenicity, clinical trials

## Abstract

Codon optimization has evolved to enhance protein expression efficiency by exploiting the genetic code’s redundancy, allowing for multiple codon options for a single amino acid. Initially observed in *E. coli*, optimal codon usage correlates with high gene expression, which has propelled applications expanding from basic research to biopharmaceuticals and vaccine development. The method is especially valuable for adjusting immune responses in gene therapies and has the potenial to create tissue-specific therapies. However, challenges persist, such as the risk of unintended effects on protein function and the complexity of evaluating optimization effectiveness. Despite these issues, codon optimization is crucial in advancing gene therapeutics. This study provides a comprehensive review of the current metrics for codon-optimization, and its practical usage in research and clinical applications, in the context of gene therapy.

## 1 Introduction

Codon optimization first appeared due to the search for an approach to increase the efficiency of expression of target proteins in bacterial cultures. The known property of degeneracy of the genetic code allows mRNA to encode the same proteins in different ways since 20 proteinogenic amino acids can be encoded by 61 codons (Welch et al., 2009). This property formed the basis of the codon optimization method, when, with the advent of genetic sequencing, it became evident that the usage of codons is non-random. Bias in codon usage occurs between different organisms, tissues, and sometimes even between parts of the same gene (Athey et al., 2017; Pouyet et al., 2017). Thus, it became clear that the selection of the most common codons deemed suitable for an organism or cell line during genetic engineering research allows significantly changing approaches to conducting experiments.

*Escherichia coli* was the first organism with an analyzed codon usage system. Knowing the sequences of anticodons and the abundance of various tRNAs in the cell, the authors identified criteria for codon optimality (Ikemura, 1981). The first criterion was high codon recognition, the second was the highest abundance of tRNA. Highly expressed genes had a bias in frequency of use towards optimal codons, while genes with low expression were characterized by high randomness in the choice of codons (Gouy and Gautier, 1982).

Currently, codon optimization has found application in a wide range of topics. In addition to fundamental research, control of the efficiency of protein expression through the selection of synonymous codons is also used for the development and production of biotherapies (Ayyar et al., 2017), most of which are based on the expression of recombinant proteins. The method has become indispensable for molecular pharming on plants, where the problem of low expression efficiency is most pressing (Perlak et al., 1991; Desai et al., 2010; Thomas and Walmsley, 2014).

**FIGURE 1:**
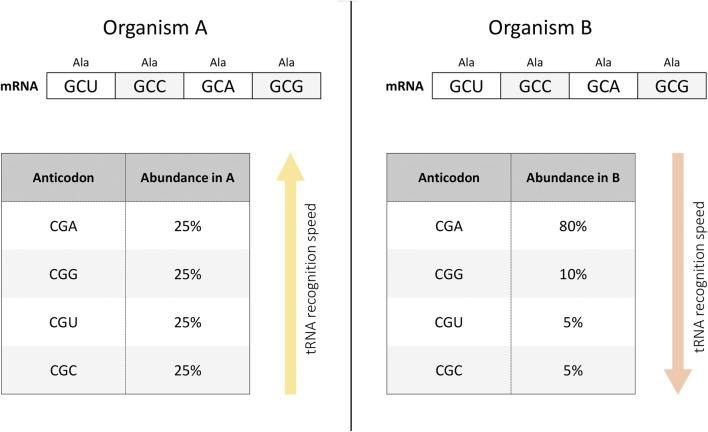
tRNA recognition depends on the abundance of the tRNA variant in the cell. For example, in organism **(A)**, tRNAs interacting with synonymous codons encoding alanine are represented in equal proportions (left panel). At the same time, it is possible that in organism **(B)**, tRNA species with different anticodons are present in a different ratio (right panel). Then, when implementing an mRNA construct with an equal frequency of use of synonymous codons encoding alanine, the rate of tRNA recognition will be higher in organism **(A)** than in organism **(B)**. In other words, the translation rate of the same mRNA construct may differ in different organisms depending on the presence of different tRNA variants.

Differentiated cells determine the formation of tissues of various types. This complicated process can be modulated at the cellular and molecular level (Simon et al., 2018). At the molecular level, this diversity is reflected in particular in differences in protein expression - proteins that are abundant in one tissue may be absent in another (Thul and Lindskog, 2018). Differences in protein abundance are, in turn, caused by differences in RNA expression. One of the possible factors affecting such patterns is the different frequency of use of synonymous codons encoding the same amino acid during translation (Kames et al., 2020) (Figure 1). Indeed, either the rarity of codon usage (Plotkin et al., 2004) or the frequency of tRNA variants (Dittmar et al., 2006; Gao et al., 2022) both vary between tissues. This can potentially be exploited for the construction of tissue-specific gene therapy. At the same time, to our knowledge, there is currently only one paper in peer-reviewed journals that has experimentally tested this hypothesis (Hernandez-Alias et al., 2023). This study is evidence that tissue-specific codon usage can potentially be used to design tissue-specific transgenes. At the same time, this metric is only one additional tool in the gene design toolbox whose implementation needs to be further explored and cannot be considered in isolation from several other indicators discussed below (Hernandez-Alias et al., 2023).

One of the most relevant and important areas of codon optimization application is the development of vaccines. The current way to create non-live vaccines is the use of attenuated viruses. Several research groups have experimented with attenuating poliovirus by changing codon bias in the gene encoding the poliovirus capsid protein, which involved replacing more frequent codons with less frequent ones (Burns et al., 2006; Mueller et al., 2006). Moreover, increasing transgene expression in vaccines may improve the effectiveness of immunization and can be achieved through codon optimization (Chen et al., 2008; Bell et al., 2016). In addition, a new class of vaccines—mRNA vaccines—has recently been introduced into clinical practice in the context of the COVID-19 pandemic (Oliver et al., 2020). Currently, the possibility of a similar approach for the prevention of infectious diseases such as rabies (Wan et al., 2023), influenza virus (Lee et al., 2023), Zika virus (Bollman et al., 2023), Lassa virus (Ronk et al., 2023) is the subject of active research and development. Remarkably, codon optimization of mRNA vaccines can significantly improve their stability and immunogenicity (Zhang et al., 2023). Despite the benefits of codon optimization, it is important to maintain a balance in the use of these techniques. Excessive interest in codon optimization can possibly lead to the accumulation of substances that are poorly excreted from the body, such as, for example, modified mRNA and the corresponding antigen (Bansal et al., 2021; Röltgen et al., 2022).

Currently, various approaches could be used for the development of gene therapeutics. Control of the immunogenicity of the administered drug is one of the most vital tasks not only in the preparation of vaccines but also for gene therapies. For the drug to work effectively, it is necessary to reduce the viral vector’s immunogenicity. It has been shown that by varying synonymous codons in the transgene and vector, it is possible to increase the effectiveness of therapy by lowering immunogenicity (Athanasopoulos et al., 2011; Bell et al., 2016), which provides optimism for simplifying vector selection and expanding the application of this type of therapy.

Regrettably, codon optimization techniques, while widely employed in the development of gene therapies, are far from perfect and are fraught with several challenges. One prominent issue lies in the incomplete synonymy of substitutions. This drawback carries the potential to disrupt natural post-transcriptional modification sites or, alternatively, give rise to novel sites, leading to critical alterations in the final protein’s structure, properties, and functions (Godfried Sie et al., 2012; Irimia et al., 2012). Furthermore, overlooking the existence of alternative translation initiation sites (Malarkannan et al., 1999; Matsuda and Mauro, 2010)can lead to the unintended production of new proteins, adding another layer of complexity to the process. Beyond these intrinsic challenges, the selection of an appropriate numerical method for evaluating the effectiveness of codon optimization poses an additional obstacle. The abundance of metrics available complicates the task, requiring careful consideration to ensure a meaningful assessment. Despite the above difficulties, codon optimization approaches are actively used in clinical trials around the world and, furthermore, COVID-19 mRNA vaccines Pfizer/BioNTech and Moderna employ codon optimization.

Codon optimization can be carried out in many different ways today. It is often not clear which of these approaches is best suited to fulfill a particular task. The purpose of this review is to cover the current state of this problem and future directions for codon optimization approaches for gene therapies.

## 2 The quantitative assessment of codon usage and optimization

### 2.1 Measures of codon usage

The codon usage bias (CUB), also known as codon usage preferences (CUP), is influenced by a combination of factors that vary among species. Such factors include mutation frequency (Pizzo et al., 2015), selection for translation efficiency (Navon and Pilpel, 2011), and the presence of transfer RNA (tRNA) molecules that recognize specific codons (Buchan, 2006; Wei et al., 2019), ribosome binding efficiency (Shi et al., 2020), and translation speed and co-translational protein folding (Mitarai et al., 2008; Liu, 2020).

Based on the non-random usage of codons in the genomes of different species and the previously demonstrated positive correlation between codon bias and gene expression efficiency, Sharp and Li developed the relative synonymous codon usage (RSCU) scale (Sharp and Li, 1986). The RSCU value was calculated for a set of genes as the ratio of the observed codon frequency to the expected frequency, assuming equal usage of synonymous codons. This research has made a substantial contribution to the creation of various metrics, including but not limited to codon adaptation index (CAI) (Sharp and Li, 1987), average ratio of RSCU (ARSCU) (Chamani Mohasses et al., 2020), and genetic tRNA adaptation index (gtAI) (Anwar et al., 2023). CAI continues to be a widely employed metric in both commercial and academic applications. CAI reflects the level of species-specific codon adaptation and is calculated as the geometric mean of RCSU values for each codon in the gene relative to the value of the most frequently used triplet encoding a single amino acid.

To date, numerous metrics for quantitative assessment of the level sequence optimization have been developed. Table 1 offers concise descriptions of commonly used metrics. To give the readers an idea of the frequency of metric usage, we added the citation rate of the original sources. However, it is important to emphasize that this approach does not reflect the level of usage of optimization tools based on the mentioned metrics.

**TABLE 1:** Metrics for codon optimization with formal definition and description. The number of citations was retrieved from the Scopus database.

Index	Mathematical formula	Principle	Interpretation	Original source	Citation count
Frequency of optimal codons (Fop)	Fop = Nopt/Ntotal, where Nopt denotes number of optimal codons in a gene, Ntotal indicates number of total codons in sequence	The rationale behind Fop is that genes with a higher expression level tend to preferentially use certain codons, and this bias can be quantified using this ratio	Representing the occurrence of the optimal codon within a gene sequence. Values close to 1 indicate a strong bias toward optimal codons, while values closer to 0 suggest a more even or random usage of synonymous codons	Ikemura, 1981 (1982)	1724 and 768
Relative synonymous codon usage (RSCU)	$RSCU=\frac{Xij}{\;\frac{1}{ni}\sum_{j=1}^{ni}\left(Xij\right)}$ where *xij* denotes the number of occurrences of the *j*-th codon for the *i*-th amino acid, *ni* represents the degeneracy for the *i*-th amino acid	Calculated as the ratio of the observed codon frequency to the expected frequency assuming equal usage of synonymous codons	The codon with RSCU of 1 value indicates average (random or equally) synonymous codon usage; RSCU value greater than 1 indicates positive codon usage bias (overrepresented); RSCU value less than 1 indicates negative codon usage bias (underrepresented)	Sharp and Li (1986)	653
Codon adaptation index (CAI)	$CAI=\exp\left(\frac{1}{L}\sum_{l=1}^{L}\ln\;W_{k}\right)$ , where *L* is the length of a gene measured in codons. Wk is the relative adaptiveness value for the k-th codon in the gene	Quantifies the geometric mean of the RSCU for each codon with respect to the codon usage of a reference set of highly expressed genes. Genes that possess higher scores are anticipated to demonstrate enhanced efficiency in translation and elevated levels of protein expression	A higher CAI score implies that the gene’s codon usage is better aligned with the preferred codons identified in a highly expressed reference set. A CAI value of 1 suggests that the codon usage in the gene is perfectly adapted to the preferred codons observed in a highly expressed reference set; value of 0 indicates that the codon usage in the gene is not adapted	Sharp and Li (1987)	4091
Codon pair adaptation index (CPAI)	$CPAI=\prod_{i=1}^{N-1}w_{i,i+1}^{1/\left(N-1\right)}$ , where N − 1 is the number of codon pairs in gene g and w-i, j is the relative adaptiveness of the codon pair (i, j). $w_{i,j}\left(G\right)=\frac{f_{i,j}\left(G\right)}{f_{\max\left(i,j\right)}\left(G\right)}$ , where *fi*, *j* is the frequency of codon pair (*i*, *j*) and *f(max(i,j))* is the frequency of the codon pair most often used to code for the amino acid pair (*aa(i),aa(j)*) in a set of highly expressed genes *G*	The advantage of the index is the automatic weight selection algorithm Carbone et al., 2003. Combined use of CPAI and CAI have been shown to better predict gene expression	Similar to CAI.	Carbone et al. (2003), Friberg et al. (2004)	43 and 402
Codon bias index (CBI)	$CBI=\frac{\left.\left(Nopt\;-Nrand\right)\right)}{\left(Ntot\;-\;Nrand\right)}$ , where *Nopt* represents the number of preferred optimal codons, *Ntot* is the total number of codons in the gene, and *Nrand* denotes the expected number of preferred codons if random codon assignments were made for each amino acid	Codon bias refers to the unequal usage of synonymous codons encoding the same amino acid in a DNA sequence. The codon bias index is a measure that quantifies the extent of this bias	Values range from 0 to 1: 0 indicates random selection of codons, and 1 indicates a significant bias towards preferred codons	Bennetzen and Hall (1982)	1955
Effective number of codons (ENc or Nc)	$ENc=\frac{1}{Faa}$ , where $Faa=n\sum_{i=1}^{k}\frac{p_{i}^{2}-1}{n-1},n>1$ , where k is the degeneracy of the amino acid (number of synonymous codons), $p_{i}$ is the fraction of each synonymous codon ( i ) out of the total codons ( n ) for that amino acid, $p=n_{i}/n$	The ENc is a measure designed to assess codon usage bias by evaluating how far the observed usage of synonymous codons deviates from an anticipated equal distribution	The ENc values range from 20 (maximum codon bias, only one codon used for each amino acid) to 61 (no bias, all synonymous codons used equally). If the ENc value is less than 35, it indicates a pronounced bias	Wright (1990)	2306
Effective number of codon-pair (ENcp)	$ENcp=\sqrt{\sum m\left(\frac{k_{m}}{F_{m}}\right)}$ , where Fm is the average degeneracy of all amino acid pairs with m synonymous codon pairs, and km is the number of amino acid pairs with m synonymous representations	ENcp is a metric designed to assess the bias in the usage of codon pairs, defined analogously to ENc, with the addition of a square root	Similar to ENc	Alexaki et al. (2019b)	100
Codon usage similarity index (COUSIN)	${COUSIN}_{18}^{a}=\frac{1}{N}\times \frac{\sum c\epsilon k_{a}\;W_{c,a}^{que}}{\sum c\epsilon k_{a}\;W_{c,a}^{ref}}$	COUSIN evaluates the codon CUB of a given query in comparison to a reference, and it standardizes the results by applying a Null Hypothesis that assumes random codon usage. In COUSIN18, all 18 families of synonymous codons contribute equally to the overall index, whereas in COUSIN59, each family contributes proportionally based on the frequency of the corresponding amino acid in the query	A COUSIN score of 1 signifies that the CUB in the query closely resemble those in the reference dataset. A COUSIN score of 0 indicates that the CUB in the query align with those in the Null Hypothesis. For scores exceeding 1, the CUB in the query share similarities with those in the reference but on a larger scale. Scores falling between 0 and 1 suggest that the CUPrefs in the query are akin to those in the reference but with a smaller magnitude. A score below 0 implies that the CUPrefs in the query are opposite to those in the reference	Bourret et al. (2019)	51
	${COUSIN}_{59}^{a}=f_{a}^{q}\times \frac{\sum c\epsilon k_{a}\;W_{c,a}^{que}}{\sum c\epsilon k_{a}\;W_{c,a}^{ref}}$ , where N is the number of amino acids present in both the query and the reference and $k_{a}$ is the set of synonymous codons coding amino acid a. weight for each codon (Wc,a) in reference and test set $f_{a}^{q}$ is the frequency of the amino acid a in the query				
Average ratio of RSCU (ARSCU)	$ARSCU=\left(\frac{\sum_{aa:1}^{aa18}\frac{a}{b}}{18}\right)$ , where aa is amino acid, a is RSCU of GC end codons and b is RSCU of AT end codons (any a and b with a value of zero is arbitrarily assigned a value of 0.1)	Measures the ratio of RSCUs with GC-ending codons to the AT-ending codons for all amino acids in a gene	Genes exhibiting ARSCU values surpassing 13 (a subjective threshold) are anticipated to showcase heightened expression levels. Genes with ARSCU values within the range of 9–13 are predicted to have either high or intermediate expression, while genes possessing ARSCU values below 9 are expected to demonstrate low or intermediate expression	Chamani Mohasses et al. (2020)	21
Relative codon bias strength (RCBS)	$RCBS={\;\left(\prod_{l=1}^{L}\left(1+d_{xyz}^{i}\right)\right)}^{\frac{1}{L}}-1,d_{xyz}=\frac{f\left(x,y,z\right)-f_{1}\left(x\right)f_{2}\left(y\right)f_{3}\left(z\right)}{f_{1}\left(x\right)f_{2}\left(y\right)f_{3}\left(z\right)}$ , where L is the length, in codons, of the gene, f (x,y,z) the observed frequency of codon xyz and f1(x), f2(y) and f3(z) the observed frequencies of bases x, y and z at, respectively, codon positions 1, 2 and 3	It calculates the observed frequency of specific codons relative to the expected frequency, considering biases in base composition at three codon sites, providing a reliable measure of codon preferences while accounting for sequence-specific features like GC content	An RCBS value near 0 implies an absence of codon usage bias, while a value exceeding 0.5 indicates a notable preference for specific codon usage	Roymondal et al. (2009)	109
Directional codon bias score (DCBS)	$DCBS=\frac{\sum_{i=1}^{L}d_{xyz}}{L},d_{xyz}=\max\left(\frac{f\left(x,y,z\right)}{f_{1}\left(x\right)f_{2}\left(y\right)f_{3}\left(z\right)},\frac{f_{1}\left(x\right)f_{2}\left(y\right)f_{3}\left(z\right)}{f\left(x,y,z\right)}\right),$	DCBS is based on RCBS and allows the measurement of both positive and negative codon usage bias	Similar to RCBS.	Sabi and Tuller (2014)	116
Modified relative codon bias strength (MRCBS)	$MRCBS=\prod_{i=1}^{N}{\left({MRCBS}_{xyz}\right)}^{\frac{1}{N}}\;{MRCBS}_{xyz}=\frac{{RCBS}_{xyz}}{{RCBS}_{aa,\max}}$ , where fxyz is the normalized codon frequency of a codon xyz and fn(m) is the normalized frequency of base m at codon position n in a gene. RCBSaa, max is the maximum value of RCBS of codon encoding the same amino acid aa in the same reference set, and N is the codon length of the query sequence	Ribosomal protein is used as a reference set of genes and the dependence on gene length is overcome	The score of the modified relative codon bias ranges from 0 to 1	Das (2017)	1
Relative codon adaptation (RCA)	${RCA}_{xyz}=\frac{f\left(x,y,z\right)}{f_{1}\left(x\right)f_{2}\left(y\right)f_{3}\left(z\right)},RCA={\left(\prod_{i=1}^{L}{RSA}_{xyz}\left(i\right)\right)}^{1/L}$ , where fxyz is the observed relative frequency of codon xyz in any reference gene set, fi(m) is the observed relative frequency of base m at codon position i in the same reference set and L is the length of the query sequence	The RCA index calculates the anticipated frequency of a codon within a provided reference set by considering the positional base frequencies. Then, it assesses codon adaptation by comparing the observed codon frequency with the anticipated frequency	The score of the RCA ranges from 0 to 2	Fox and Erill (2010)	107
Codon deviation coefficient (CDC)	The calculation of the metric can be found in the original source	The index takes into account the nucleotide composition of the sequence, GC content, and purine content. CUB is estimated using the cosine distance between the expected and observed codon usage vectors. Assessing the statistical significance of results using bootstrap resampling	Values range between 0 and 1: value 0 - no bias, 1 - maximum bias	Zhang et al. (2012)	62
Index of Translation Elongation (ITE)	$I_{TE}=\frac{\sum_{e^{i=1}}^{N_{s}}F_{i}\ln\;w_{i}\;}{\sum_{i=1}^{N_{s}}F_{i}},w_{i}=\frac{S_{i}}{\mathrm{Max}\left(S_{i}\right)}$ , where $F_{i}$ is the frequency of codon i , $N_{s}$ is the number of sense codons (excluding those in single-codon families)	CAI-like index, but it involves determining a weight for each codon by considering its frequency within the NNR and NNY codon subfamilies in the reference set. Subfamilies are distinguished due to different translation by different tRNAs and susceptibility to different mutational errors	ITE values range between 0 and 1. A greater score is assigned to genes containing codons that are more commonly found in highly expressed genes	[25]	136
Synonymous codon usage order (SCUO)	$SCUO=\sum_{i=1}^{n_{i}}\left(\frac{\sum_{j=1}^{n_{i}}x_{ij}}{\sum_{i=1}^{18}\sum_{j=1}^{n_{i}}x_{ij}}\right){SCUO}_{i,}\;{SCUO}_{i}=\frac{H_{i}^{\max}-{\;H}_{i}}{H_{i}^{\max}}$ where *j* is the codon *i*-th amino acid. *SCUOi* is the SCUO for *i*-th amino acid in each sequence and *Hi* and *Hmaxi* are the entropy and maximum for an *i*-th amino acid in a sequence	The SCUO index assesses how much a sequence deviates from a uniform distribution, using Shannon entropy as a basis. It involves the normalized difference between the maximum entropy and the observed entropy	SCUO the value varies between 0 and 1, and higher values indicate a stronger codon usage bias	Wan et al. (2006)	21

**TABLE 2:** Example representation of the 4-letter amino acid sequence ADGY (alanine-aspartic acid-glycine-tyrosine) via synonymous codons. Nucleotide sequence of wild-type GCC-GAT-GGT-TAT. There are 4 codon variants for the first and third amino acids, and 2 variants for the second and fourth amino acids. Total 64 possible variants of nucleotide presentation of this sequence.

Synonymous codon variants	1st	2nd	3rd	4th
Amino acid				
**A**	GCT	GCC	GCA	GCG
**D**	GAT	GAC		
**G**	GGT	GGC	GGA	GGG
**Y**	TAT	TAC		

Numerous metrics can be easily calculated with a reference set of genes to obtain the codon usage frequency. For example, Fop is calculated as the ratio of optimal codons to the total number of codons, excluding stop codons and codons without alternatives for amino acids (methionine, tryptophan) (Ikemura, 1981; 1982). The index aids in gauging the prevalence of synonymous codon usage. Other metrics are grounded in the assumption that the usage of codons is non-random. The metrics quantify the difference in codon usage frequency from a uniform distribution within the coding sequence. When all codon variants for a specific amino acid are utilized with equal frequency, such difference is minimal. Conversely, the maximum is achieved when only one codon out of the possible ones is utilized. Examples of such indices include ENC, CDC, SCUO, and others.

### 2.2 Codon adaptation metrics for assessing mRNA properties

Codon optimization is a strategy aimed at increasing the efficiency of mRNA translation and overcoming protein expression limitations. The use of synonymous codons affects the stability of mRNA in human cells (Narula et al., 2019; Wu et al., 2019). The thermodynamic stability of mRNA within a cell significantly influences translation efficiency (Hanson and Coller, 2018; Diez et al., 2022). mRNA is inherently unstable and can undergo transient states and adopt multiple stable structures. One approach to selecting synonymous amino acids for the purpose of thermodynamic stabilization is aimed at minimizing the free energy ΔG (MFE) released during RNA folding (Zuker and Stiegler, 1981; Zuker, 1994). Ringner and Krogh demonstrated in *Saccharomyces cerevisiae* that the folding free energy in the vicinity of the 5′-UTR correlates positively with transcription efficiency and mRNA half-life (Ringnér and Krogh, 2005).

An alternative approach suggests that the optimal structure will possess the maximum number of chemical bonds (Wayment-Steele et al., 2021). The AUP (Average Unpaired Probability) and SUP (Sum of Unpaired Probabilities) metrics, employed to assess RNA stability against hydrolytic degradation, operate under the premise that structures formed by paired bases exhibit lower susceptibility to hydrolysis.

Cluster analysis discovered that different mRNAs preferentially use different types of codons. Some mRNAs predominantly use optimal codons, while others prefer non-optimal codons. Furthermore, they observed that mRNAs with a higher proportion of optimal codons tend to be more stable, while those with a lower proportion of optimal codons are more unstable. Based on conducted experimental research, a metric called the codon stability coefficient (CSC) has been proposed. It is calculated as the Pearson correlation coefficient between the frequency of each codon and mRNA half-lives (Presnyak et al., 2015).

In the standard genetic code, the first two positions of a codon play a decisive role in coding an amino acid, while the third position is variable for one amino acid. Collection of metrics developed GC1, GC2, and GC3 represents the frequency of G + C usage at the first, second, and third positions, respectively (Stenico et al., 1994). Another evaluation derived from RSCU is the Average RSCU Ratio (ARSCU) (Chamani Mohasses et al., 2020). Its noteworthy feature involves considering the base at the third position of the codon. The optimization of protein expression often involves the frequent usage of GC content. The model of post-transcriptional mRNA regulation involving P-bodies, 5′-3′ exonuclease XRN1, RNA helicase DDX6, and enhancer of decapping PAT1B shows that GC-rich coding sequences (CDS) result in higher protein production compared to AU-rich ones, and are controlled by a mechanism involving degradation factors DDX6 and XRN1 (Courel et al., 2019). On the contrary, reducing the GC content in the 5′-UTR leads to an increase in free energy and also enhances protein yield, presumably due to mRNA destabilization in the translation initiation region and greater accessibility of the ribosome binding site (Dewi and Fuad, 2020). The GC3 content varies depending on the type of tissue but is not an exhaustive characteristic for tissue-specific gene separation (Plotkin et al., 2004). GC3 codons are also associated with a longer half-life of mRNA (Kudla et al., 2006; Hia et al., 2019).

### 2.3 Metrics for adaptation to tRNA pool

Codon usage bias is closely linked to translational selection, which is the process of selecting codons that match abundant tRNAs, the molecules responsible for carrying amino acids during protein synthesis. Highly expressed genes tend to use such preferred codons, resulting in enhanced translation rates and accuracy. Dittmar et al., 2006 showed that the expression levels of nuclear and mitochondrial tRNAs vary between human tissues, indicating tissue-specific translational selection. However, minor differences in mouse mitochondrial RNA have only been detected for cardiac tissue, while significant differences between the central nervous system and other tissues have been demonstrated at the level of tRNA isodecoders, i.e., transcripts with the same anticodon but encoded by numerous different genes (Pinkard et al., 2020). It is important to note that the strength of translational selection varies across different organisms based on their genome sizes and genomic tRNA content (Reis, 2004).

To account for the role of intracellular tRNA content in translation efficiency, the following indices have been developed: P2index (Gouy and Gautier, 1982) and tRNA adaptation index (tAI) (dos Reis, 2003).

Initially, tAI was only applicable to *S. cerevisiae*, but its subsequent modifications, stAI (Sabi et al., 2017) and gtAI (Anwar et al., 2023)—overcome this limitation by incorporating species-specific weights through algorithmic approaches to find extrema. gtAI demonstrated greater efficiency by employing a genetic algorithm to identify the optimal set of weights. In its calculation, indices ENc and RSCU are also incorporated. gtAI ranges from 0 to 1, where a higher value implies better adaptation of the codon to the tRNA pool.

The P2 Index is a metric used for the quantitative assessment of the efficiency of interactions between codons and their corresponding anticodons during the translation process. Based on the frequency of specific types of codons, values exceeding 0.5 indicate the presence of translational selection influencing the coding sequence.

### 2.4 Algorithmic approaches and tools for codon optimization

Currently, various optimization algorithms are utilized, such as the genetic algorithm (Błażej et al., 2018), multi-objective artificial bee colony (Gonzalez-Sanchez et al., 2019), Ribotree Monte Carlo (Leppek et al., 2022), and dynamic programming (Pham et al., 2004; Taneda and Asai, 2020), to identify codon combinations with desired characteristics. In several studies, the use of recurrent neural networks for codon optimization in heterologous protein expression has been presented in Chinese hamster (*Gricetulus griseus*) ovary cells (Goulet et al., 2023) and *E. coli* (Jain et al., 2023). The Bidirectional Long Short-Term Memory (LSTM) deep learning model has also been trained for *E. coli* (Fu et al., 2020).

Other studies applied machine learning methods for mRNA stabilization, such as integrated deep learning-based mRNA optimization (iDRO) (Jain et al., 2023), which provides a two-step optimization for the open reading frame and the untranslated regions. S. Castillo-Hair and G. Seelig trained a model on the 5′UTR polysome profile dataset to predict ribosome loading and protein expression (Castillo-Hair and Seelig, 2022). The predictive power of such models strongly depends on the quantity and quality of the training datasets. At the same time, the accumulation of experimentally verified data sets is often not as fast as the development of machine learning methods. For example, to date (February 2024) only 6,142, of which 1,416 are human, experimentally validated RNA structures have been deposited in the Protein Data Bank (Berman, 2000). This indicates that the high-precision prediction of RNA 3D structures using machine learning methods may be accurate for training data, but not for new data (Sato and Hamada, 2023).

Several software tools that utilize statistical and algorithmic solutions are available for commercial and free use. Here, we present some current tools that can be used for various tasks, including those related to gene therapy: ATGme (Daniel et al., 2015), OPTIMIZER (Puigbo et al., 2007), CHARMING (Wright et al., 2022), %MinMax (Rodriguez et al., 2018), JCat (Grote et al., 2005), Optipyzer (LeRoy and Roleck, 2023), IDT (Owczarzy et al., 2008), gtAI (Anwar et al., 2023).

## 3 Codon optimization for gene therapy vectors

Above, the elucidation of metrics and principles related to codon optimization has been expounded. At the same time, it should be noted that the resources required to test the functionality of *in silico* predicted RNA variants significantly exceed the cost of the prediction itself. For this reason, studies often mainly present unconfirmed hypotheses in *in vitro* or *in vivo* experiments. Nevertheless, we present below some examples where codon optimization has been successfully applied *in vitro*. Proceeding to *in vitro* studies, it should be noted that gene therapeutics consist of a delivery vector and a therapeutic gene. Currently many types of vectors are used as a transgene vehicle (e.g., lipoplexes (Chen et al., 2016), polyplexes (Hayat et al., 2019), virus-like particles (Pitoiset et al., 2017)).

Some of these vectors are a cassette with the selected viral genes, others do not contain nucleic acids. In some cases, wild-type viral genes in the gene therapy vector are not optimized for efficient application (Bainbridge et al., 2008). At the same time, codon-optimized variants of these sequences increase the efficacy of gene therapy, although they may lead to unfavorable results such as undesirable conformational changes and subsequently alterations in protein activity and function. Examples of codon optimization of adenoviral (Coughlan, 2020), retroviral and lentiviral vectors (Breckpot et al., 2010) are discussed below.

Since adeno-associated vectors have recently become the most widely used platform for gene transfer (Mendell et al., 2021) and adenoviruses have long been successfully used to deliver genes (Bulcha et al., 2021), we will consider the application of optimizations on their example.

It has been shown that in adenoviruses, the genes responsible for highly abundant late structural proteins tend to use codons frequently used in humans (optimal codons), while early regulatory use less optimal codons (Villanueva et al., 2016). However, the adenoviral fiber protein specifically uses suboptimal codons for efficient viral replication. Surprisingly, analysis of transgenes expressed in oncolytic adenoviruses, that are used for the oncoselective expression of a wide range of therapeutic molecules (de Sostoa et al., 2019; Huang et al., 2019) shows that most transgenes also use suboptimal codons. This contradicts the recommendation to use optimal host codons in transgenes to maximize gene expression. The study investigates the impact of transgene codon usage on viral fitness and finds that transgenes with higher GC3 content (optimal codon usage) have higher gene expression and viral replication, while those with lower GC3 content have lower expression and replication (Núñez-Manchón et al., 2021). By tuning the codon usage of transgenes, it is possible to achieve better transgene expression without compromising viral replication, thus optimizing the therapeutic outcome.

In the development of gene therapies, the problem arises of achieving high titers and a high ratio of empty to full capsids in viral vectors. One of the solutions to this obstacle is codon optimization of viral genomes encoding capsid proteins and assembly proteins. Thus, not only transgenes but also the coding sequences of the viral vector itself are subjected to codon optimization. For AAV-based (adeno-associated virus) vectors a novel codon optimization method was presented (Localized Codon-Optimization or LCO) (Cabanes-Creus et al., 2019).

This method aims to preserve functional elements of the capsid genes and improve capsid shuffling efficiency for AAV engineering. The LCO algorithm performs localized optimization of codons at each position independently, based on the usage frequency of codons observed in the input variants of AAV sequences. A codon usage frequency table is generated for each amino acid position, and this table is used to optimize individual sequences (Table 3). The LCO-modified capsid genes showed increased sequence identity between parental AAV capsids and novel AAV capsid variants.

**TABLE 3:** An example of how the LCO method works to optimize the four codons of the mRNA encoding ADGY (see Table 2). A probability is calculated for all possible codons for a particular amino acid at a particular position. The most probable codons are marked in bold. Accordingly: GCC-GAT-GGT-TAT (wild-type nucleotide sequence)—would be optimized to GCT-GAT-GGA-TAC (final LCO-optimised sequence).

Probability of finding a codon at a given position				
codon	Position 1	Position 2	Position 3	Position 4
GCT	**0.4**			
GCC	0.17			
GCA	0.24			
GCG	0.19			
GAT		**0.68**		
GAC		0.32		
GGT			0.26	
GGC			0.22	
GGA			**0.36**	
GGG			0.16	
TAT				0.39
TAC				**0.61**

Functionality tests demonstrated that the optimized capsids retained their function, and transduction efficiency was similar to unoptimized counterparts. The LCO method also improved the efficiency of capsid shuffling, resulting in a highly shuffled library with increased complexity and reduced size of donor sequence segments. The shuffled clones generated using LCO-encoded capsids demonstrated successful transduction, indicating the effectiveness of LCO in generating novel AAV variants.

Ironically, the extensive use of codon optimization occurred simultaneously with abundant research findings that revealed the impact of synonymous mutations on protein function. This has been shown on a variety of proteins (Buhr et al., 2016; Kirchner et al., 2017).

The mechanism being discussed involves the comparison between codon-optimized (CO) and wild-type (WT) variants of a protein named FIX (coagulation factor IX). The results highlight that the CO and WT FIX variants exhibit distinct conformations, suggesting that the codon optimization process has influenced the protein’s structure. Ribosome profiling analyses uncover altered ribosomal distribution patterns and local translational kinetics in the CO variant when compared to the WT variant. Notably, these differences are unique to the CO FIX variant, as control genes demonstrate comparable ribosome distribution profiles (Alexaki et al., 2019a).

Despite the observed differences in translational kinetics, the overall efficiency of protein synthesis between the CO and WT variants remained similar. This finding is consistent with previous studies conducted *in vitro* (outside of a living organism) and suggests that the rate of protein synthesis is comparable between the two variants. The researchers propose that differences in translational kinetics within these domains may contribute to the observed conformational differences between the CO and WT FIX variants.

Codon optimization can be approached not only by a global view of codon usage in general, but also by a local optimization for each individual position in a particular amino acid. Moreover, it is also important to check that the functions of the essential elements and the optimized protein of interest remain unchanged.

## 4 The effect of codon optimization on immunogenicity

**FIGURE 2:**
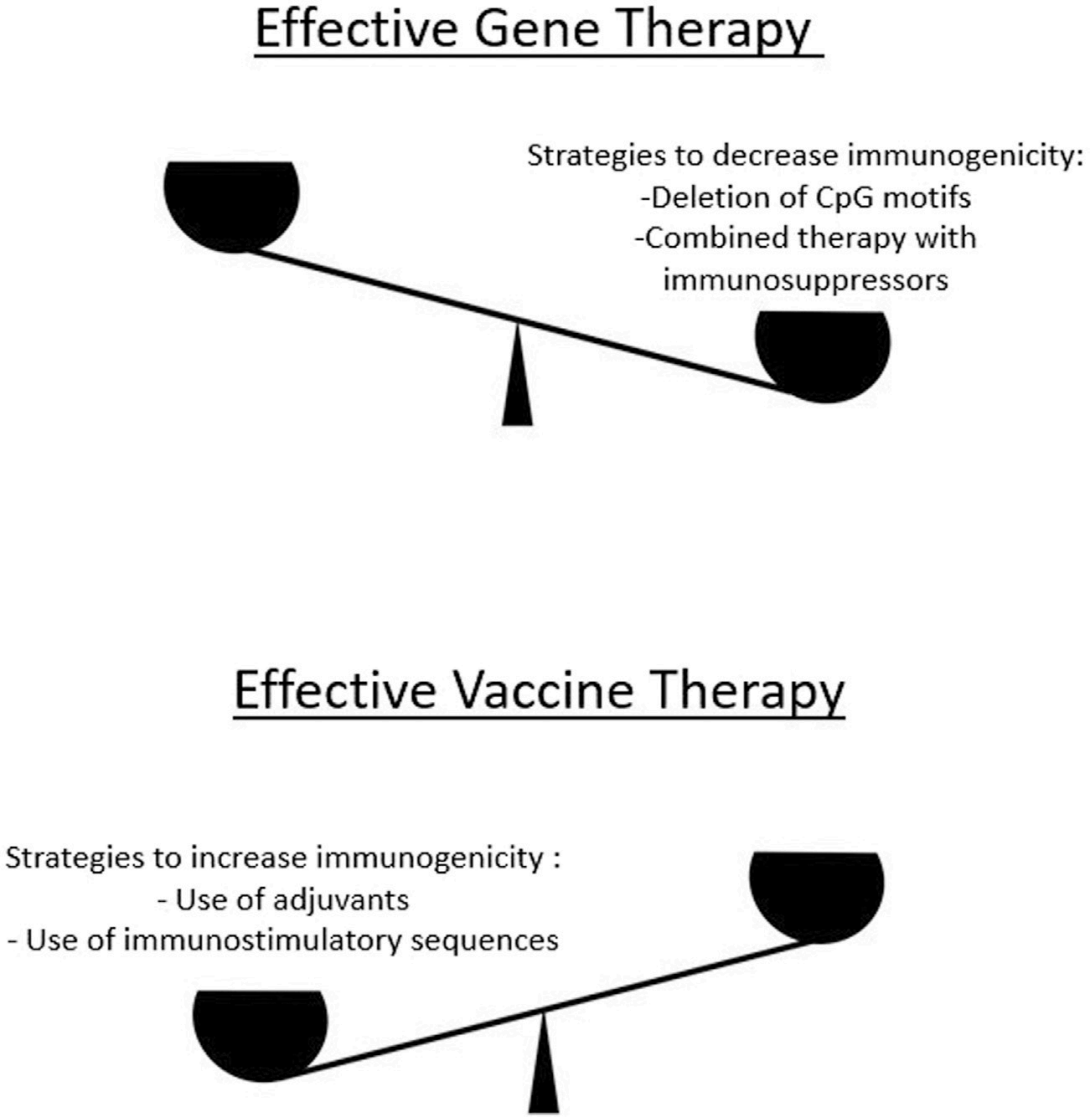
To develop effective gene therapies, a delicate balance must be maintained in terms of increasing or decreasing immunogenicity. On the one hand, excessive immunogenicity reduces the efficacy of a gene therapy product because less protein is produced in the corresponding tissues. Therefore, there are approaches to reduce excessive immunogenicity (upper panel). On the other hand, for certain classes of gene therapy products that target the development of an immune response (e.g., mRNA vaccines), methods are used to increase immunogenicity (lower panel).

The immune response to an administered foreign substance or molecule can be defined as immunogenicity. It should be noted that higher immunogenicity increases the efficacy of the drug in some cases, but decreases it in others (Figure 2). For example, the purpose of immunization is to generate an immune response against a pathogen. In this case, methods should be used to increase the immunogenicity of the drug. It should be noted that in the development of mRNA vaccines, an excessive overreaction of the immune system is undesirable due to possible damage to the human organism (Igyártó and Qin, 2024) and should be taken into account during codon optimization. On the other hand, if a transgene introduced into the organism is intended to lead to the production of the corresponding protein, any degree of immunogenicity will reduce the effectiveness of the therapy. The innate and adaptive immune response to gene therapy may vary depending on the source of immunogenicity. These may be factors related to the capsid of the virion or to the viral genome. In relation to the capsid, binding of TLR2 or TLR9 can potentially activate the innate immune response and initiate the MyD88 signaling cascade, which in turn stimulates the production of proinflammatory cytokines such as TNF-alpha or induces the synthesis of IFN-gamma (Yang et al., 2022). Depending on the composition of the viral vector, the innate immune response can lead to enhanced adaptive immune responses. For example, AAVs, which are often used as gene therapy vectors, circulate naturally between humans. As a result, most people develop antibodies against natural AAV serotypes due to previous exposure. These antibodies are even known to cross-react with engineered vectors (Boutin et al., 2010). As a result, these antibodies can lead to either complement activation or neutralization of the capsid. The adaptive immune response is characterized by the degradation of the capsid protein by the proteasome and peptide presentation on MHC class I molecules. CD8^+^ cytotoxic T-cell lymphocytes can bind to the MHC, which leads to cell death (Martino et al., 2013). Peptide presentation on MHC class II molecules after phagocytosis and proteolysis can be recognized by CD4^+^ T lymphocytes, which can then stimulate the proliferation of B cells and the production of capsid-specific antibodies (Li et al., 2013). Studies have shown that plasmacytoid dendritic cells (pDCs) and conventional dendritic cells (cDCs) co-operate to achieve cross-priming of CD8^+^ T cells (Rogers et al., 2017). pDCs recognize the AAV genome via TLR9, while cDCs present the antigen on MHC I. The binding of cytokine-produced IFN to its receptor on cDCs is necessary for this process, indicating a direct relationship between pDC-produced cytokines and the activation of cDCs. Cross-priming of CD8^+^ T cells against AAV capsids requires CD40^−^CD40L co-stimulation, which is performed in addition to T1 IFN from CD4^+^ Th cells (Shirley et al., 2020b).

After viral uncoating, TLR9 receptors can recognize unmethylated CpG motifs in the released single-stranded DNA, which also leads to activation of the innate immune system and stimulates cytokine production. The humoral and cellular innate immune responses described above for AAV capsids also occur for the transgene protein. The adaptive immune response can depend on various factors such as the target tissue, vector design and dose. Depending on the specificity of the promoter, there is a potential risk of immunogenicity (Shirley et al., 2020a). For example, a ubiquitous promoter can increase the risk of an adaptive cellular immune response of target and non-target cells (Sun et al., 2005).

It should be noted that the appearance of a foreign protein in the human organism is associated with the development of autoimmune diseases due to the similarity of individual epitopes of foreign and self proteins (Rojas et al., 2018). For example, it was recently shown that the same antibodies cross-react with the Epstein-Barr virus protein and the human alpha-crystallin B protein (Thomas et al., 2023). This phenomenon of molecular mimicry could be associated with the development of multiple sclerosis. The possibility of molecular mimicry of proteins resulting from the translation of the nucleic acids used must therefore be taken into account in the development of gene therapeutics. As already mentioned, codon optimization of the RNA can influence the structure of the translated protein (Alexaki et al., 2019a). As a result, depending on the different variants of the synonymous substitutions, the presentation of different epitopes of the same protein is possible.

It is of interest to reduce these CpG motifs to circumvent the possible human immune response, which can be achieved by codon optimization. For example, various elements of an AAV vector such as the CMV enhancer and promoter, ITR regions, UTR regions and the therapeutic transgene itself may contain CpG motifs. The CpGs within the promoter sequence can be removed, but with unpredictable effects on the activity and specificity of the promoter. For example, the authors have shown that the removal of CpGs within the CMV promoter gene significantly reduces its activity (Yew and Cheng, 2004). Although CpGs can be removed from the expression cassette, as in the case of human coagulation factor IX (hFIX) (Bertolini et al., 2021), this does not always increase efficiency—CpG elimination had only reduced antibody formation against the transgene and not against the capsid itself. There are several studies in which this strategy was used, but mostly with a modification of the transgene. They have shown that the elimination of CpG motifs may lead to a significant reduction in the CD8^+^ T cell response (Yew and Cheng, 2004; Faust et al., 2013; Herzog et al., 2019; Wright, 2020; Bertolini et al., 2021; Konkle et al., 2021).

Several codon optimization strategies, including the chemical modification of nucleosides (Karikó et al., 2005) and the incorporation of pseudouridine (Karikó et al., 2008; Anderson et al., 2010; Thess et al., 2015), have been shown to improve translation and reduce the immune response to mRNAs. pDCs exposed to such modified RNA exhibit a significant reduction in cytokines and activation markers. Nucleoside modification at a single position in a chemically synthesized oligoribonucleotide (ORN) is sufficient to abrogate TLR activation. In addition, the incorporation of pseudouridine in particular has been shown to facilitate evasion of recognition by Toll-like receptors (Karikó et al., 2005), although the molecular differences contributing to this mechanism has not yet been elucidated. Although the implementation of pseudouridine increases the stability of the mRNA and its translational capacity, it is important to note the disadvantages of replacing uridine with pseudouridine (Xia, 2021; Mueller, 2023). A recent study has shown that the presence of pseudouridine in IVT mRNA increases ribosomal + 1 frameshifting during mRNA translation. In addition, new peptides were generated that triggered an immune response (Mulroney et al., 2024). The presence of pseudouridine in the stop codon region suppresses translation termination and allows non-canonical base pairing, which is particularly detrimental for *in vitro* transcribed mRNAs (Loomis et al., 2016). The negative effects of pseudouridine synthases have been associated with various cancers (Xue et al., 2022) and autoimmune diseases (Festen et al., 2011). This strongly suggests that the influence of codon optimization and pseudouridine incorporation on mRNA expression needs to be further investigated. A limitation of the present review is that it does not focus on a detailed description of the specific effects of codon optimization on the mRNA vaccines against COVID-19 *per se* that have been introduced into clinical practice (reviewed in Xia, 2021), but aims to discuss the advantages and disadvantages of the different options for the use of codon optimization in gene therapy in general.

To summarize, a common strategy to avoid immunogenicity is to eliminate redundant CpG motifs, implement chemical modifications of ORNs and replace uridine with pseudouridine. However, it should be noted that the implementation of codon optimization to eliminate CpG motifs and pseudouridine modification must be performed strategically to avoid the negative consequences of both approaches. Given the various unresolved factors leading to potential immunogenicity as a consequence of gene therapy, developing metrics for prediction is a complicated task. Nevertheless, a recent report (Wright, 2020) proposed a metric for prediction focusing exclusively on CpG motifs and their potential immunogenicity. Three formulas were developed that take into account the amount of unmethylated CpG motifs in the vector sequence. Known immunostimulatory sequences commonly used in DNA vaccines were also considered in the development of the formulae (Bode et al., 2011). Although these formulae still need to be improved for full validation and accurate prediction, they reflect the beginning of a deeper understanding of how codon optimization can contribute to the reduction of immunogenicity.

## 5 Experimental testing of codon optimized sequences

**FIGURE 3:**
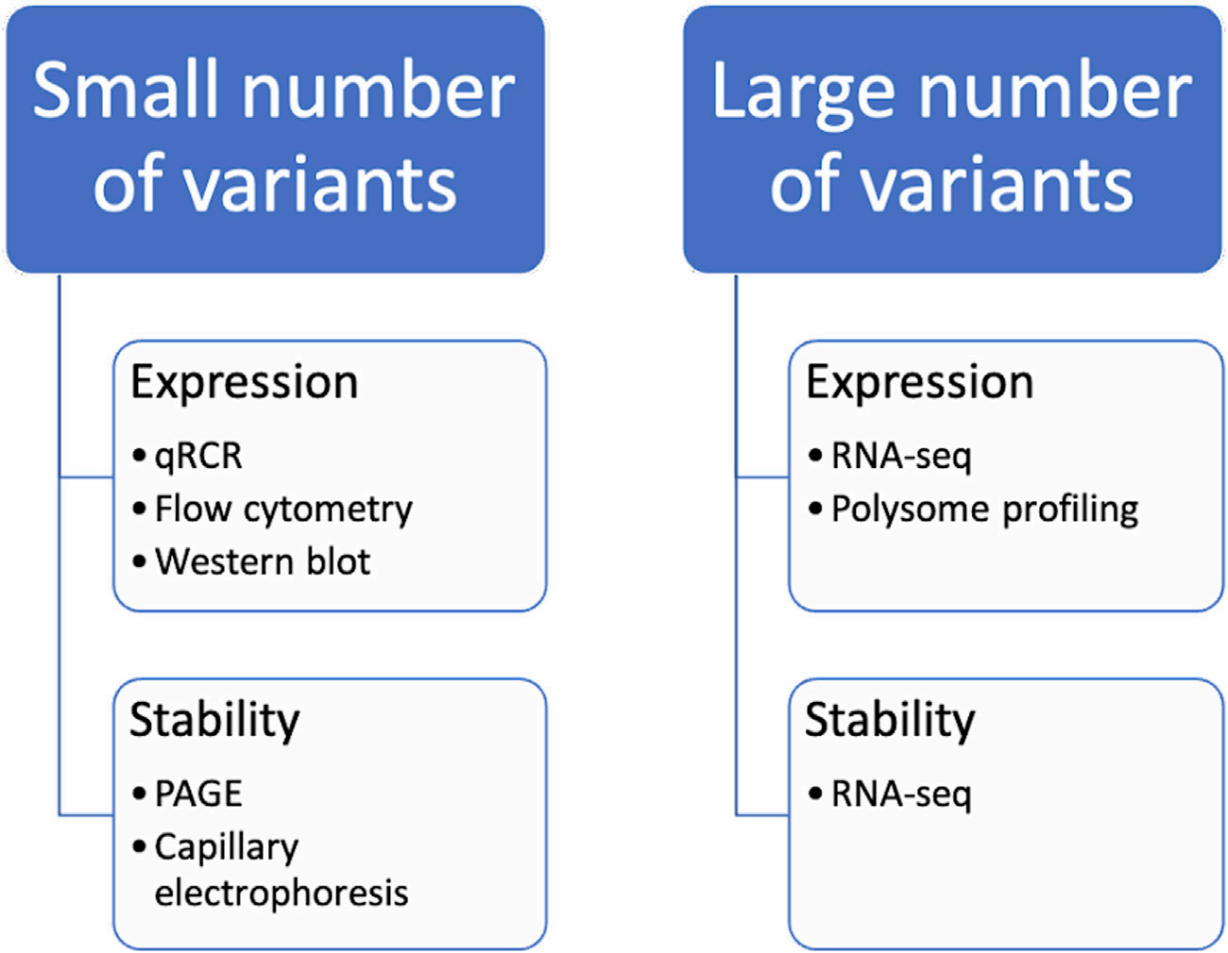
Methods for the analysis of codon-optimized sequences. It should be noted that when studying the properties of a small number of variants of mRNA constructs, certain methods of analysis are used, while when comparing a large number of variants of mRNA constructs at the same time, others are used.

There are numerous strategies for optimizing codons in nucleic acids. The methods mentioned above enable the creation of numerous optimized sequence variants. However, experimental verification of properties such as mRNA stability and protein expression levels is necessary before further experimentation can be conducted. Depending on the goals and available resources, it may be possible to select the best candidates based on chosen criteria from the range of design variants. These candidates can then be examined using routine laboratory methods. Alternatively, a pool of hundreds of sequences can be studied, in which case high-throughput protocols must be developed (Figure 3).

When studying a small number of variants, it is possible to determine the expression level separately for each construct after transfecting the cells. To quantify transgene expression in this case, the most common method is to use target-specific primers with cDNA obtained from RNA by reverse transcription as a matrix and perform qPCR (Leppek et al., 2022). Expression can be quantified at both the transcriptional and translational levels. The latter involves the analysis of synthesized proteins and can be performed using antibodies specific to the target protein. For instance, Zhang (Zhang et al., 2023) described the properties of the optimized structure of the SARS-CoV-2 virus S protein using flow cytometry. A possible alternative method for determining protein concentrations is to use SDS-PAGE gels for Western blot analysis, along with specific antibodies (Raab et al., 2010; Fath et al., 2011).

Although codon optimization of the target sequence can provide certain benefits, it may also result in reduced mRNA stability in solution, which impairs its functionality. Therefore, it is necessary to experimentally confirm the stability of the structure of optimized nucleic acids. The stability of mRNA molecules is inversely proportional to their degradation rate in solution. To determine the degradation rate, mRNAs are incubated in PBS buffer containing Mg2+ ions. Samples are collected at various time intervals of 1–2 h, and the number of fragments produced is estimated using capillary electrophoresis (Zhang et al., 2023) or polyacrylamide gel electrophoresis with urea. Therefore, the RNA is less stable if it degrades more quickly after being incubated in solution.

However, the laboratory approaches described above are time-consuming when testing multiple variants of codon-optimized sequences. In light of this, there is a great need to create high-throughput methods for studying many sequences simultaneously.

Most methods that allow mass screening of sequences follow a general principle: a unique barcode, a sequence of several nucleotides, is inserted into each variant. All the sequences to be tested can then be pooled and processed in a multiplex format. The presence of the barcode makes it possible to identify a variant using high-throughput sequencing platforms after all the necessary protocol steps have been completed.

Massively parallel variant analysis requires the synthesis of a library of DNA templates. The next steps in the study can be performed in two ways. The first involves transcription and modification (3′ polyA tail and 5′ m7G capping) *in vitro*, followed by transfection of the resulting mRNA pool into cells for further experiments. The “PERSIST-seq” method was developed based on this approach. It enables the simultaneous evaluation of stability and translation efficiency of over 200 mRNA molecules, making it a convenient tool for messenger RNA development (Leppek et al., 2022). In this case, the design of the DNA must take into account the presence of a promoter in the initial sequence. The second approach involves creating a vector library with cassettes that contain the sequence under study and regions of homology. The cells are then transfected with the library, and the sequences are integrated into the genome using CRISPR/Cas. This process enables the direct synthesis of mRNA within the cells. A study of the motifs that cause ribosome slowdown in a yeast model system describes a similar approach (Chen et al., 2023). The next steps for experimental validation in both cases involve isolating RNA from cell culture, analyzing it through high-throughput sequencing, and quantifying the results. To identify inserts in the pool of isolated nucleic acids, unique barcodes are introduced into the library construct, which is a common aspect of the described strategies.

The presence of unique barcodes in the original DNA matrices allows quantitative assessment of the expression level for each individual variant using high-throughput RNA sequencing.

Translation of sequence variants has been demonstrated to be a crucial determinant in mammalian gene expression (Burke et al., 2022). However, genomic expression profiling alone cannot reveal the precise regulation provided by post-transcriptional mechanisms, such as 5′ capping, splicing, polyadenylation, nuclear export, translation, and decay. To overcome this limitation, a polysome profiling method can be used to isolate ribosome-free and polysome-associated RNAs for further independent analysis (Pereira et al., 2018) This method involves separating mRNA in a sucrose gradient into two fractions: polysome-bound and polysome-free. The mRNA is then isolated from both fractions and sequenced using one of the available high-throughput platforms.

When studying multiple variants, stability assessment is also important. To identify full-length molecules that have not degraded, it is necessary to amplify the cDNA that was reverse transcribed from the RNA and then sequence it to quantify the amount of intact mRNA at each time point. This method can evaluate mRNA stability in both solution and cells. The solution replicates the conditions in which the molecules may be present during therapy, typically high pH and positively charged media. It is important to note that the outcomes obtained after incubation in solution differ significantly from those obtained after isolation from cells. This is likely due to cellular mechanisms of RNA degradation (Leppek et al., 2022).

Therefore, there are approaches that allow for the evaluation of the efficiency and stability of nucleic acid sequences obtained during codon optimization. The choice of a particular method depends on the number of variants to be analyzed. If there are only a few variants, it is possible to describe the properties of each variant separately, providing a fairly accurate understanding of its characteristics. When dealing with hundreds or thousands of variants, high-throughput methods are necessary. This allows for a pool of samples to be tested instead of individual samples, greatly increasing the productivity of experimental work. It is important to note that massively parallel sequencing methods provide high accuracy analysis, while polysome profiling can offer additional insights into the impact of codon optimization on the final product’s quality.

## 6 Future directions

Currently, there are some gene therapies that use different codon optimization metrics and are approved by the FDA (FDA, 2024). To analyse other therapies that are in clinical trials and where codon optimization has been used, we conducted a thorough examination of the data available on ClinicalTrials.gov (ClinicalTrials.gov, 2024) until December 2023. A systematic search strategy was devised using the keyword “gene therapy” in the Condition/disease field. In addition to the specified search criteria, it is important to note that the term “vector” was included in the “Other terms” considered in the search. The algorithm did not include any specified values for the “Intervention/treatment” and “Location” categories in the search process. After searching, the algorithm automatically incorporated synonyms for the given query: gene: “Genes,” gene therapy: “Gene transfer”; “Gene Transfer Procedure,”, therapy: “treatment”; “Therapeutic”; “therapeutics”.

Furthermore, a comprehensive search was conducted using the specific only Condition/disease of “codon optimized” and excluded any specified values for the “Other terms,” “Intervention/treatment” and “Location” categories in the search process. However, it is crucial to mention that studies explicitly referring to monoclonal antibodies and enzymes as drugs in the Study URL and Brief Summary columns were manually excluded from the sample. This careful exclusion strategy ensured that the selected studies focused specifically on codon optimization. The search was conducted over a period of 20 years to capture an extensive range of relevant clinical studies.

**FIGURE 4:**
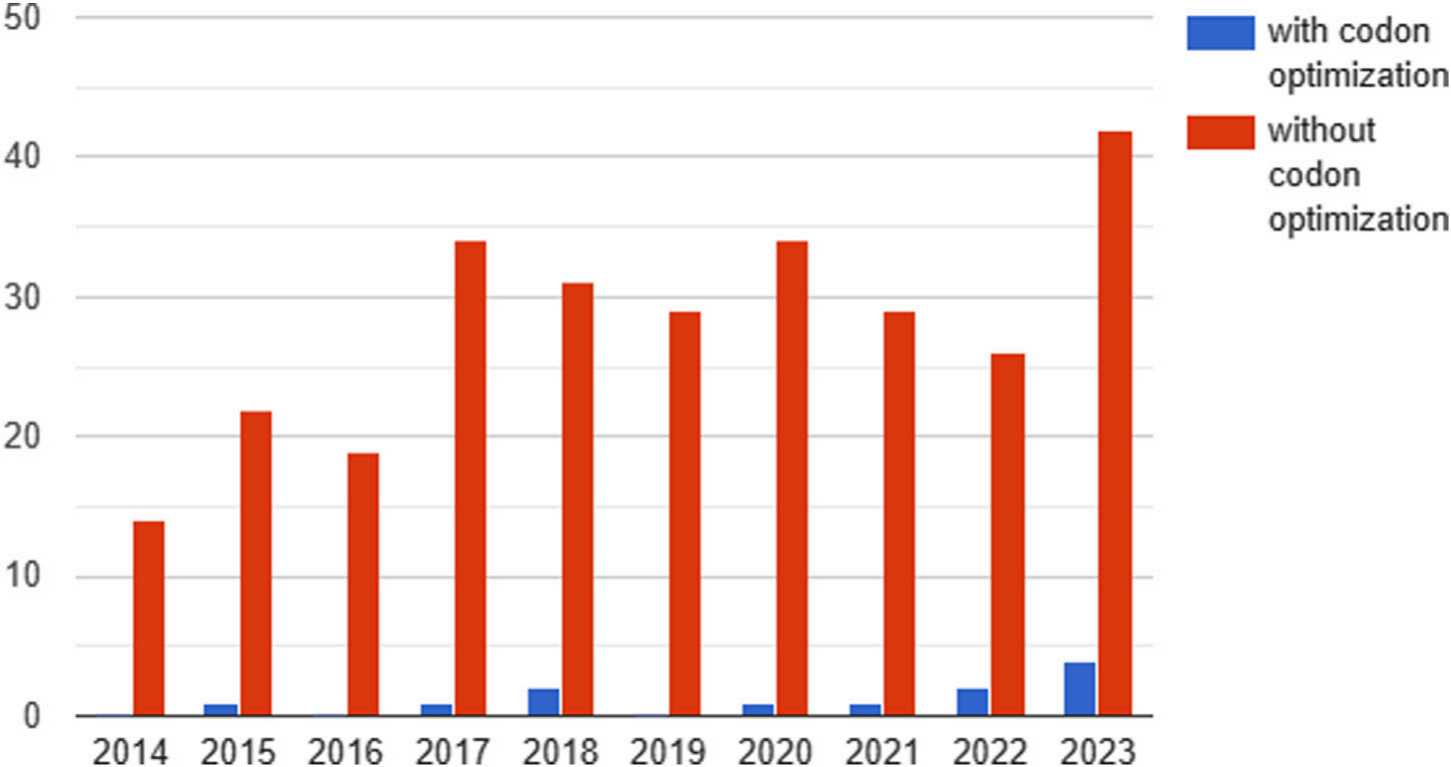
Dynamics of the number of studies reported on clinicaltrails.gov testing gene therapeutics with and without codon optimization by year (2014-2023). Since 2020, a trend towards an increase in the proportion of studies with codon optimization can be observed.

Of the 395 clinical studies analyzed, only 12 contained information on codon optimization (Figure 4).

Prior to experimental testing of codon-optimized sequences using any of the aforementioned methods, it is essential to synthesize these sequences, often in large quantities. The most widely used method currently is phosphoramidite synthesis, which involves the interaction of nucleotide phosphoramidite monomers protected by acid-labile groups with an activating agent, binding to the growing oligonucleotide (Sinyakov et al., 2021). There are two main types of implementation for this approach, depending on the equipment used: synthesis on columns or on microarrays. The former option allows for the synthesis of oligonucleotides at a relatively low cost and with an error rate of 1 per 600 base pairs or less on average. However, it does not provide sufficient throughput for mass synthesis of oligonucleotides (Ma et al., 2012). Furthermore, if the sequence of interest exceeds 200 base pairs (some estimates suggest 300 (Palluk et al., 2018)), an additional assembly step via molecular cloning is required (Casini et al., 2015). These factors significantly limit the speed of testing and represent the primary bottleneck in experimental design.

This problem can be solved by integrating higher-throughput oligonucleotide microarray synthesisers into laboratory practice (Song et al., 2021). Commercially available technologies are also based on phosphoramidite synthesis, albeit with slight modifications. Although microarray-based nucleotide synthesis is more error-prone due to heterogeneity and edge effects, it enables the synthesis of oligonucleotide pools and also reduces the cost per nucleotide by 2–4 orders of magnitude compared to column synthesis (Kosuri and Church, 2014). This suggests that advances in *de novo* DNA synthesis and experimental verification of codon-optimized sequences are likely to be associated with the microarray approach.

Since 2020, a trend towards an increase in the proportion of codon-optimized studies has been observed. In 2020, 1 in 34 (2.9%) clinical trials used codon optimization, compared to 4 in 42 (9.5%) in the first 11 months of 2023 (Figure 4). The main aim of codon optimization was to increase the level of transgene expression and the stability of the mRNA. In addition, a study using codon optimization to reduce immunogenicity was reported in 2021.

To effectively achieve the goals of codon optimization in research, it is important to follow established metrics. However, today there is no single generally accepted standard for codon optimization. Therefore, it is possible to use a large number of combinations of the methods described above to create optimal RNA variants. Some of these approaches significantly increase the efficacy of gene therapeutics. Therefore, several drug options have been registered in clinical trials, for example.

Codon optimization has played an important role in the development of RNA-based COVID-19 vaccines. Current research efforts are focused on further advancing the field of codon optimization for COVID-19 vaccines to address new strains of the coronavirus (Wu et al., 2023). Unfortunately, it was not possible to provide here the specific metrics used for codon optimization in the above-mentioned studies for commercial product development. This limitation results from the intellectual property of the original codon-optimized constructs. In this article, we have explored various metrics for assessing codon usage, based on both the composition of the coding sequence and the composition of a reference set of genes. One widely used metric is the Codon Adaptation Index (CAI). Although these measures provide useful information about adaptation to the host organism, they do not necessarily indicate an increase in translational efficiency due to selection pressure (Rahman et al., 2018; Feng et al., 2022). Furthermore, CAI is also interpreted as an indicator of the speed of translational elongation (Kudla et al., 2009). In turn, an increase in translation speed may not necessarily result in the production of a protein with similar properties in greater quantities.

**FIGURE 5:**
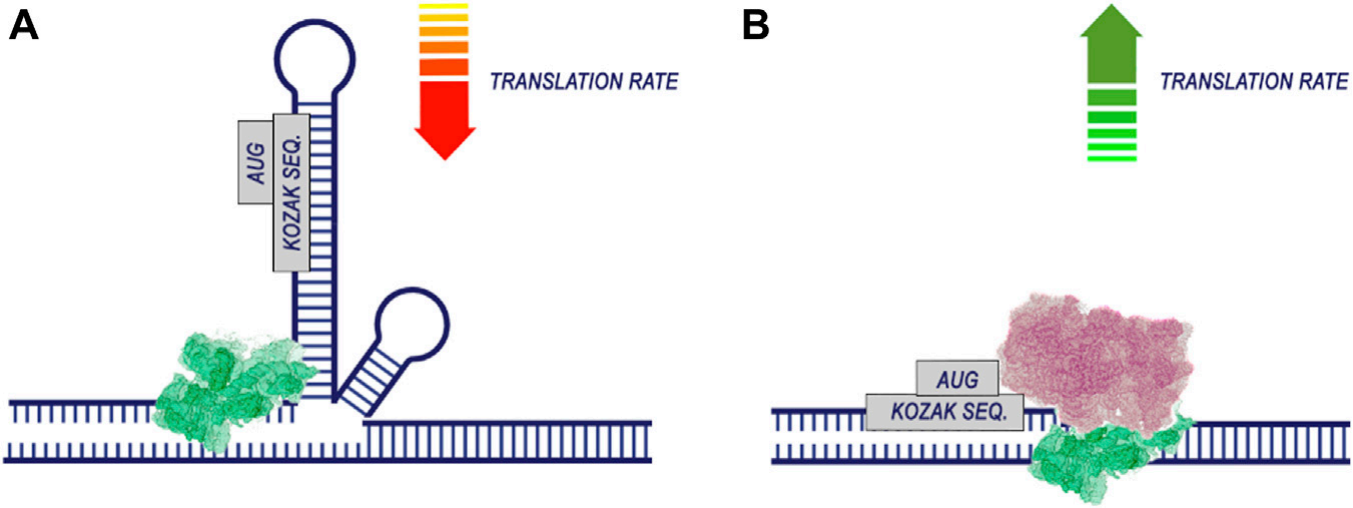
The secondary structure of RNA reduces the efficiency of translation. The process of translation initiation is completed by the recognition of the start codon by the 43S preinitiation complex and the assembly of the ribosome. If the region of the start codon is hidden in the secondary structure of the RNA **(A)**, translation is likely to be less efficient. At the same time, if there are no pronounced secondary RNA structures in the region of the start codon **(B)**, the probability of translation initiation increases.

Apparently, during translation, the most important regions for codon optimization are the areas around the start codon. This is supported by work demonstrating the contribution of the CDS position near the start codon (Höllerer and Jeschek, 2023; Nieuwkoop et al., 2023) and the 5′UTR sequence region (Capell et al., 2014). The efficiency of translation is significantly dependent on the energy of mRNA folding, particularly in the vicinity of the start codon (Gu et al., 2010). This is associated with the fact that unfolding more stable RNA secondary structures require greater energy before the initiation of translation (Figure 5). Additionally, the presence of hairpin, stem-loop, and pseudoknot structures in mRNA can hinder ribosome translocation and tRNA binding, thus impeding translation elongation (Kozak, 2005; Bao et al., 2020).

Thus, advancements in gene therapy could be directed towards a more comprehensive exploration of the impact of codon optimization on the characteristics and secondary structure of mRNA.Also, it is possible to apply optimization metrics locally to the start region, but there are limitations since many of them are based on codon usage frequency without taking into account the features of untranslated regions.

In addition, consideration of local codon optimization is a critical aspect that must be taken into account during codon optimization for a particular protein of interest. Furthermore, essential protein functions may change due to the possible influence of codon optimization on the conformation of the resulting protein, which should also be taken into account.
